# Contribution of Known Genetic Risk Variants to Dyslipidemias and Type 2 Diabetes in Mexico: A Population-Based Nationwide Study

**DOI:** 10.3390/genes11010114

**Published:** 2020-01-20

**Authors:** Alicia Huerta-Chagoya, Hortensia Moreno-Macías, Magdalena Sevilla-González, Rosario Rodríguez-Guillén, María L. Ordóñez-Sánchez, Donají Gómez-Velasco, Liliana Muñóz-Hernández, Yayoi Segura-Kato, Olimpia Arellano-Campos, Ivette Cruz-Bautista, Carlos A. Aguilar-Salinas, Teresa Tusié-Luna

**Affiliations:** 1 CONACYT, Instituto Nacional de Ciencias Médicas y Nutrición Salvador Zubirán, Mexico 03940, Mexico; ahuerta@conacyt.mx; 2 Unidad de Biología Molecular y Medicina Genómica, Instituto Nacional de Ciencias Médicas y Nutrición Salvador Zubirán, Mexico 14080, Mexico; 3 Departamento de Economía, Universidad Autónoma Metropolitana, Mexico 09340, Mexico; 4 Clinical and Traslational Epidemiological Unit, Massachusetts General Hospital, Boston, MA 02114, USA; 5 Departamento de Endocrinología y Metabolismo, Instituto Nacional de Ciencias Médicas y Nutrición Salvador Zubirán, Mexico 14080, Mexico; 6 Departamento de Medicina Genómica y Toxicología Ambiental, Instituto de Investigaciones Biomédicas, UNAM, Mexico 04510, Mexico

**Keywords:** cholesterol, HDL, LDL, triglycerides, dyslipidemias, type 2 diabetes, Mexicans, genetics, polygenic risk score

## Abstract

Dyslipidemias are common risk factors for the development of chronic disorders including type 2 diabetes (T2D). Over 100 associated *loci* have been identified but few reports have evaluated the population attributable fraction captured by them in population-based nationwide surveys. Therefore, we determined the population contribution of a set of known genetic risk variants to the development of dyslipidemias and T2D in Mexico. This study included 1665 participants from a Mexican National Health Survey carried out in the year 2000. It is a probabilistic complex sample survey of households, which comprises representative data at a national level. 103 previously reported SNPs associated with different dyslipidemias or T2D were genotyped and used to compute polygenic risk scores. We found that the previously known variants associated with dyslipidemias explain at most 7% of the total risk variance of lipid levels. In contrast, the known genetic risk component for T2D explained a negligible amount of variance (0.1%). Notably, variants derived from the Native-American ancestry have the strongest effect and contribute with a high proportion of the variance. These results support the need for additional studies aimed to identify specific genetic risk variants for Mexican population.

## 1. Introduction

Mexico has experienced an epidemiological transition that went from a communicable disease predominance to a non-communicable chronic disease (NCCD) pattern, which mainly affects the adult population [1]. In 2014, NCCD were responsible for 77% of the mortality rate in Mexico [2]. Specifically, cardiovascular diseases were the leading cause of NCCD deaths [2] and dyslipidemias were the most common risk factor. In the year 2000, the prevalence of the three most important dyslipidemias in Mexican adults was 63% for hypoalphalipoproteinemia, 49% for hypertriglyceridemia and 43% for hypercholesterolemia [3], meanwhile, type 2 diabetes (T2D) prevalence was 7.5% [4]. According to the World Health Organization (WHO) estimates, in 2012 people living in the Eastern Mediterranean region, followed by the region of the Americas, had the highest probability of dying from a NCCD. Lack of lipid determinations representative to all the regions of the world has prevented to give global estimates of the prevalence of dyslipidemias. However, as reported in Mexico, cardiovascular diseases account for most NCCD deaths worldwide. Similarly, WHO reported that in 2012 the prevalence of diabetes was higher in the Eastern Mediterranean region, followed by the Americas, the Western Pacific, and the South East Asia regions, the last three having comparable estimates. In all cases, European populations showed the lowest estimates of diabetes and deaths caused by NCDD [5].

Like other metabolic disorders, dyslipidemias and T2D are heritable traits and their genetic component comprises a set of common variants with typically low effect sizes and different prevalence among human populations. Different studies have identified over 100 human *loci* associated with lipid levels and a similar number for T2D, but the proportion of trait variance explained by currently known common genetic variants fluctuate from 10 to 30% [6,7,8]. Most of these studies have focused on genetically homogeneous cohorts from European populations. However, it is well known that ethnicity is associated with large differences in the prevalence of the risk alleles, leading to different contributions of each variant to the burden of disease. Moreover, recent studies using admixed Latino cohorts have successfully narrowed European lipid and T2D *loci* and helped to prioritize candidate genes [9,10,11,12].

Although there are different methods to quantify how much of the underlying genetic basis of disease is explained by known risk loci, the polygenic risk scores (PRS) aim to capture the overall variance in a trait conferred by the set of genetic variants grouped into a composite score. In this study, we computed a PRS composed of variants at the extreme of a statistical distribution, i.e., restricted-to-significant association on the trait of interest (rsPS) [13].

## 2. Materials and Methods

The aim of this study was to determine the population contribution of a set of known genetic risk variants to the development of dyslipidemias and T2D in samples derived from a Mexican population-based nationwide survey, which is free of potential bias that may alter the strength of the associations under study.

### 2.1. 2000. National Health Survey

The 2000 Mexican National Health Survey (NHS-2000) is a national cross-sectional probabilistic survey that was conducted between the years 1999 and 2000 in Mexico. Through a multi-stage cluster sampling design 1470 households per state for a total of 47,070 households nationwide were selected. Into each household one child, one teenager and one adult 20 years or older were randomly selected. NHS-2000 provided representative data at different levels: national urban and rural areas and the 4 regions in the country (South, North, Center and Mexico City and Metropolitan area). A detailed description of the sampling framework and methodology has been previously published [14]. In the present study, we used a subpopulation of non-related adult men and women.

Demographic data and medical history were recorded using a standardized questionnaire. Blood pressure was measured with the subject in the supine position after 5 min rest. Height and body weight were measured on a daily-calibrated balance. Waist circumference was measured at the midpoint between the highest point of the iliac crest and the lowest part of the costal margin in the mid-axillary line. Trained personnel obtained blood samples, which were used to purify DNA and to separate serum. Serum was frozen at −50 °C until metabolic profiling. Determinations of serum total cholesterol (TC), high-density cholesterol (HDLc), low-density cholesterol (LDLc) and triglycerides (TG) were performed through Synchron CX Delta (Beckman Coulter) colorimetric enzymatic methods at the Departamento de Endocrinología y Metabolismo of the Instituto Nacional de Ciencias Médicas y Nutrición Salvador Zubirán (INCMNSZ). For the purpose of this study, only individuals who had a 9 to 12 h fasting period at the moment of the blood collection were selected (*n* = 1665). Diagnosis of T2D was done following the American Diabetes Association criteria, i.e., fasting plasma glucose values ≥126 mg/dL, current treatment with a hypoglycemic agent, or casual glucose values ≥200 mg/dL. As previously reported, this subsample was randomly distributed; no bias was detected for sex, education, region or socioeconomic status from the whole sample. Consequently, non-fasting and fasting subsamples had no relevant differences that could contribute to bias in the interpretation of biochemical cardiovascular risk indicators [15]. Informed consent was obtained from all participants before they participated in the study. The study was conducted in accordance with the Declaration of Helsinki, and the protocol was approved by the Ethics and Research Committees of all institutions (Protocol identification code: 1033). All samples, including DNA and serum, were stored in a Biobank at the INCMNSZ.

### 2.2. Genotyping and Quality Control Procedures

Genomic DNA was extracted from buffy coats obtained from whole blood using the QIAmp 96 DNA Blood Kit. Purity and concentration were obtained using a NanoDrop ND 1000. DNA samples were genotyped at LGC Genomics (LGC, Beverly, MA, USA). Single Nucleotide Polymorphisms (SNPs) with a call rate <97% were considered technical failures at the genotyping facility and were automatically deleted before further quality control. We directly genotyped 75 common genetic variants previously associated to dyslipidemias in several populations [9,16,17]. Quality control was performed with PLINK [18]. It included the exclusion of samples with 10% or more missing data within the full dataset. In addition, variants with 5% or more missing data, Minor allele frequency (MAF) <0.01 and monomorphic variants were also removed. All SNPs were tested for Hardy Weinberg equilibrium (*p* < 0.00001). Linkage disequilibrium (LD) between SNPs located within the same gene was computed. SNPs that failed to pass the prior tests were excluded for further analyses. After quality control, whole dataset included 1627 individuals and 98 SNPs (Table S1).

### 2.3. Polygenic Risk Score

For SNP selection, a literature search was performed. It encompassed articles published up to 2017. We prioritized SNPs that had been associated to lipid or diabetes traits at the accepted level of genome-wide significance (*p* > 5 × 10^−8^), replicated either through Genome Wide Association Studies (GWAS) or a candidate gene approach in Mexican population, as well as those with reported or genotyped allelic frequencies larger than 5% in Mexicans.

For the lipid traits, we included variants previously published by the worldwide Global Lipid Genetics Consortium (GLGC) [16]; meanwhile, for diabetes trait, we analyzed variants previously published in a metanalysis including several international Consortia [19] and replicated in the Slim Initiative in Genomic Medicine for the Americas (SIGMA) T2D Consortium, the largest project aimed to characterize the genetic basis of T2D in Mexican population [10]. Additionally, three SNPs, previously described as Native American private T2D (*SLC16A11* rs75493593 and *IGF2* rs149483638) and low HDLc (*ABCA1* rs9282541) risk genetic variants were included [10,12,17]. Neither odds ratio (OR) threshold nor allelic prevalence limits were established since they might be largely influenced by ethnic characteristics of Mexican population.

A restricted-to-significant polygenic score (rsPS) was computed based on the assumption of an additive genetic effect. The rsPS was calculated by accounting for the number of risk alleles present per SNP and summing the results over the whole sets of SNPs, using PLINK [18]. We used a weighted score, in which the effect sizes were computed on an independent Mexican sample from SIGMA Consortium including 8000 individuals [10]. A detailed description of SIGMA Consortium sample has been published elsewhere [10]. In the case of lipid traits, linear models were adjusted for sex, age, body mass index (BMI), diabetic status, PC1, and PC2. For the T2D trait, logistic models were adjusted for sex, age, BMI, PC1, and PC2. In both cases, β coefficients-specific for each trait of interest- were extracted and used as scores for rsPS computations.

### 2.4. Population Stratification Control

Mexican is a recently admixed population with both European and Amerindian ancestries as the major ethnic contributors. A principal components analysis was performed using a set of 32 Ancestry Informative Marker (AIM) genotypes, with EIGENSTRAT [20]. The applied set of AIMs was selected and previously validated from a high density GWAS data in Mexican population [21]. The top two principal components were used as covariates for correcting for population stratification, as they accounted for most of the total variance.

Additionally, we stratified the sample according to individual global ancestry proportion. It was estimated using data from the 1000 Genomes Project [22] and the Mexican Genome Diversity Project [23] as parental populations. We selected 95 non-related individuals from the European Utah population (CEU); plus 38 Mexican Native individuals (Maya and Zapoteca Native groups). The whole dataset was analyzed with ADMIXTURE [24] at *K* = 2.

### 2.5. Statistical Analyses

As part of the NHS-2000 methodology and in order to represent all eligible adults from the population, expansion weights were both calculated and modified according to the non-response and post-stratified to calibrate for population distribution. Thus, the weight of each participant in the sample signifies the number of adults that he or she represents in the population (9). Accordingly, the quality-controlled set of 1627 individuals are representative of 1,920,66, about 3.6% of the Mexican adult population 20 years or older in the year 2000.

All the analyses were performed using the package survey for the analysis of complex survey samples, in R [25]. We considered the expansion weights at the individual level, calculated for the NHS-2000. First, we conducted a descriptive analysis of the sample and their respective estimation to the whole Mexican population. Weighted allele frequencies were computed using contingency tables for survey data.

Then, we calculated the proportion of the trait variance explained by the whole set of genetic variants, as well as by the environmental variables including sex, age, BMI, ancestry. Specifically, for lipid traits, T2D status was also included. To accomplish the above, linear regression models were adjusted: model 1) regressing the trait of interest on the rsPS without adjustments and model 2) regressing the trait of interest on the rsPS adjusted for the environmental covariates. For each trait, the proportion of the variance in the outcome predictable from the genetic component was estimated using the coefficient of determination (r^2^) obtained from the model 1. In addition, the environmental variance was estimated by the difference between the r^2^ obtained from the two models (model 2-model 1) (Figure S1). To estimate the contribution by each single SNP, regression models of individual SNPs risk scores were fitted and adjusted for the environmental covariates. In all cases, regression models for complex surveys were used.

In addition, to assess if the proportion of the trait variance explained by the genetic component was modified by the gender, we generated a bootstrapped 95% confidence interval for r^2^ based on 1000 replications of regression models including either women or men only. Differences between them were probed using a t test. Also, for descriptive purposes, we divided the subjects into the four quartiles of Native American ancestry. The distribution of the rsPSs were compared among them.

Finally, we used STRING [26] to infer functional links among the proteins encoded by the genes that explained most of the genetics of lipid levels and T2D status. The enriched Gene Ontology (GO) biological processes were summarized with REVIGO [27] and plotted in a treemap using R. The enriched KEGG pathways were used to map the genes analyzed in this study.

## 3. Results

**Figure 1:**
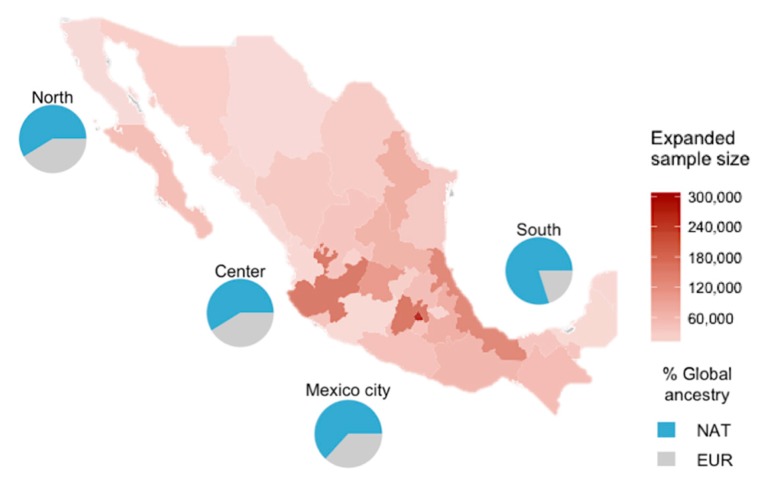
Sample size representation across states of Mexico. Pie plots show the global ancestry proportions for each of the four geographic regions of Mexico.

Sample included 1627 individuals across all country. Eligible samples had complete questionnaires, serum samples, and DNA. On average, subjects were 35.2 ± 14 years old and both men and women groups were equally represented. Based on the individual expansion weights from the NHS-2000, they represented 1,920,663 Mexican adults (3.6% of the 52,850,503 Mexican adult population 20 years or older in the year 2000) (Table 1). All states of Mexico were represented (Figure 1).

When analyzing the global ancestry proportions, we found that the individuals born in the northern region of Mexico showed lower Native American ancestry proportions, as compared to the individuals born in the rest of the country (survey *t* test, *p* < 0.001). In this study, the Native American ancestry proportions of the four geographic regions of Mexico were: North region 52.8%, Center region 58.6%, Mexico City region 63.3% and South region 79.9% (Figure 1).

Specifically, diabetic subjects were older and displayed characteristics associated with metabolic syndrome phenotype such as higher levels of systolic blood pressure, BMI, waist circumference, glucose, total cholesterol, and triglyceride levels. As expected, diabetic subjects showed a significantly higher Native American ancestry proportion than euglycemic group. As for subjects with dyslipidemia, they were older and showed higher levels of systolic blood pressure BMI, waist circumference and glucose. However, no significant differences were found in Native American ancestry proportion as compared with normolipidemic subjects (survey t or rank sum tests, *p* < 0.05, Table 2).

**Figure 2:**
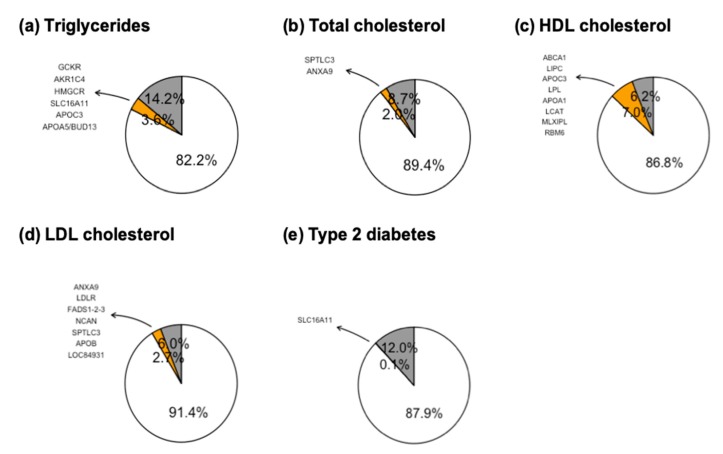
Contribution of common single nucleotide polymorphism (SNPs) to the genetic variance of lipid levels in Mexico. It is shown the variance explained by previously associated genes (yellow), as well as by environmental covariates (grey): age, sex, body mass index (BMI), ancestry and type 2 diabetes (T2D), specifically for lipid traits. In addition, the most associated genes with lipid levels (**a–d**) and T2D (**e**) in Mexican population are shown.

Five out of the 103 genotyped SNPs (75 for lipids and 28 for T2D) were excluded of the analyses given that showed a minor allele frequency lower than 0.01 (*TMEM161* rs75557067, MAF = 0.002; *APOC3* rs138326449, MAF = 0, *LDLR* rs2228671, MAF = 0.002 and *HNF1A* rs483353044, MAF = 0) or showed a high LD with another SNP within the same gene (*TCF7L2* rs7903146: r2 rs7903146 vs rs12255372 = 0.498) (Table S1). The overall genetic variance jointly explained by the 71 dyslipidemia-related genetic variants was 3.6% for TG, 2% for TC, 7% for HDLc and 2.7% for LDLc. On the other hand, the overall genetic variance explained by the 27 T2D-related genetic variants was 0.1%. Except for HDLc, the total genetic variance was greater than the environmental variance explained by sex, age, BMI, ancestry -and diabetes status, specifically for dyslipidemias (Figure 2a–e). Interestingly, the genetic variance explained by the rsPS for TG and HDLc levels were statistically higher in men (TG: 7.2% men vs. 2.7% women; HDLc: 10.1% men vs. 6.4% women). Meanwhile, the genetic variance explained by the rsPS for TC and LDLc levels were higher in women (TC: 1.7% men vs. 3.3% women; LDLc: 2.1% men vs. 5.4% women) (*p* < 0.05) (Figure S2).

Among the most influential genes for TG levels in Mexican population are *GCKR* rs1260326, *AKR1C4* rs1832007, *HMGCR* rs12916, *SLC16A11* rs75493593, *APOC3* rs4520 and *APOA5*/*BUD13* rs964184 (*p* < 0.05). In turn, *SPTLC3* rs3645585 and *ANXA9* rs267738 genes are the main contributors of TC and LDLc levels (*p* < 0.05). In addition, for LDLc levels, *LDLR* rs9305020, *FADS1-2-3* rs174546, *NCAN* rs2228603, *APOB* rs7575840 and *LOC84931* rs2030746 were among the most relevant signals (*p* < 0.05). For HDLc levels, genes with strongest associations were *ABCA1* rs9282541, *LIPC* rs1077835, *APOC3* rs5128, *LPL* rs12678919, *APOA1* rs2070665, *LCAT* rs16942887, *MLXIPL* rs2286276 and *RBM6* rs2013208 (*p* < 0.05) (Figure 2a–e and Table S2).

Remarkably, private Native American genetic variants were among the most influential for both dyslipidemias and T2D risk traits. That is the case of *ABCA1* rs9282541 which showed the highest genetic contribution to the variance of HDLc levels and *SLC16A11* rs75493593 which was among the main contributors for TG levels and T2D traits (Tables S2 and S3).

Genes that were found to have a great influence on lipid levels (*p* < 0.05) in Mexican population were functionally implicated with three biological processes (GO): (i) lipid metabolism and homeostasis, (ii) organic hydroxy compound transport and (iii) plasma lipoprotein particle organization. The main KEGG pathways enriched were: (i) cholesterol metabolism, (ii) PPAR signaling and (iii) fat digestion and absorption. Genes having greatest influence on cholesterol metabolism were *ABCA1*, *APOA1*, *APOB*, *APOC3*, *LCAT*, *LDLR*, *LIPC,* and *LPL*. Specifically, *ABCA1*, *APOA1* and APOB genes showed to be also important in fat digestion and absorption pathway. In turn, relevant genes related with PPARG signaling pathway included *APOA1*, *APOA5*, *APOC3*, *FADS2,* and *LPL* (Figure S3).

**Figure 3:**
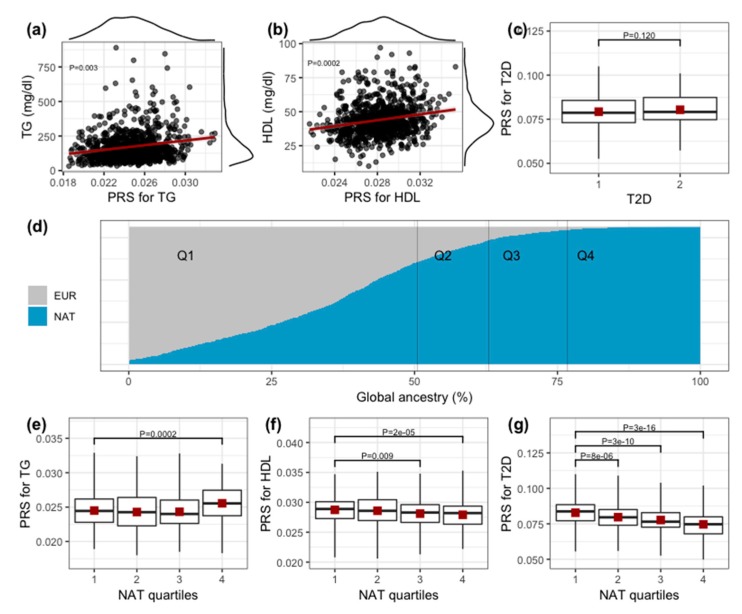
Relation between restricted-to-significant polygenic score (rsPS), its own trait level and ancestry. (**a–b**) show the relation between triglyceride (TG) and high-density lipoprotein (HDL) rsPS and its own lipid levels, as well as their density distribution. *p* value is adjusted for sex, age, BMI, type 2 diabetes (T2D) and ancestry. (**c**) shows the distribution of T2D rsPS between normoglycemic and diabetic individuals, *p* value is adjusted for sex, age, BMI and ancestry. (**d**) shows the individual global ancestry proportion of the studied sample. It was calculated from the two main parental populations of Mexicans, namely European and Native American ancestries. (**e**–**g**) show the distribution of TG, HDLc and T2D rsPS among the Native American quartiles of the studied sample. Unadjusted *p* values.

Regarding the rsPSs, we found significant associations between them and its corresponding TG and HDLc levels after adjustment for sex, age, BMI, diabetes status and ancestry (TG *p* = 0.003, HDLc *p* = 0.0002). Neither significant association was found between TC and LDLc rsPS and their corresponding lipid levels, nor of T2D rsPS with the development of the disease (TC P = 0.054, LDLc *p* = 0.089, T2D *p* = 0.120) (Figure 3a–c), suggesting that risk scores -constructed using known loci previously reported for other populations- cannot be readily applied to Mexican individuals. Interestingly, rsPS showed to be dependent on the Native American global ancestry proportion. For instance, individuals within the fourth quartile of Native American global ancestry proportion showed a significant higher rsPS for TG levels, but a lower rsPS for HDLc levels (*p* < 0.05) (Figure 3d–f). In addition, although T2D rsPS was not statistically different between normoglycemic and diabetic individuals, those with higher Native American ancestry showed a lower rsPS for T2D (*p* < 0.05) (Figure 3c,g). Given that we specifically used effect sizes computed on an independent Mexican sample, the above finding may imply differences in allelic frequencies among populations, highlighting the relevance of diversifying the genetic study of human populations.

## 4. Discussion

This is the first study assessing the contribution of known risk variants for dyslipidemias and T2D in a population-based nationwide sample from Mexico. We estimated that the known genetic risk variation for lipid traits contributes with 7%, 3.6%, 2.7%, 2%, of the variance for HDLc, TG, LDLc and, TC, respectively. In contrast, the known genetic risk variation for T2D contributes with less than 1% of the variance.

Such a small amount of variance explained for diabetes risk might be due to the fact that for T2D trait, we only analyzed 27 variants. They were previously published as part of metanalyses that included several international Consortia and were further replicated in the SIGMA T2D Consortium study -the largest project aimed to characterize the genetic basis of T2D in Mexican population. In SIGMA study, 9.2 million SNPs in 8214 Mexicans and other Latin Americans were analyzed [10]. The prior evidence in SIGMA study was used to select those variants with the largest contribution to T2D risk in Mexicans, as the analyzed variants showed an association *p* value < 0.05. It is important to mention that in the present study, we showed that T2D subjects had a significantly higher Native American ancestry proportion than the euglycemic group (58.2% euglycemia vs. 64.8% diabetes, *p* < 0.05). Hence, Non-European variation could be underestimating the explained disease risk, partially because causal variants might be in imperfect linkage disequilibrium with genotyped variants.

It is also well known that dyslipidemia is a risk factor for T2D and the prevalence of dyslipidemia in Mexico is notably high, as compared to other world regions [5]. In our population-based nationwide sample, we did not find significant differences in the prevalence of dyslipidemia status between euglycemia and T2D groups (70.6% in euglycemia vs. 78.7% in T2D, *p* > 0.05), suggesting that the known associated genes to dyslipidemias are exerting a similar influence in both euglycemic and diabetic groups.

Over the past years, many GWAS have been performed for dyslipidemias and T2D. Although only a small percentage of the total variance has been explained by the genetic component, these discoveries have directed towards new disease mechanisms and targets for prevention and therapy. However, as this study suggests, the Mexican population contribution explained by the known genetic variation is only partially shared with Caucasians. It is important to note that the frequencies of known risk variants differ significantly between Mexicans and other populations, and that the observed effects of risk alleles can also vary across populations.

For instance, in this study, the most influential genetic contribution for TG levels is attributed to *GCKR*, *AKNR1*, *HMGCR*, *SLC16A11*, *APOC3* and *APOA5/BUD13* loci. It is interesting to note that for some variants such as *APOA5/BUD13* rs964184 -consistently replicated in other populations from distinct ethnicities- [28], the risk allele frequency found in Mexicans is much higher as compared to other populations (f risk allele G: 36% in this study, 16% in Europeans, 22% in Africans and 24% in East Asians) [22]. Consistent with a previous study, *SLC16A11* rs75493593, a Native American-derived variant, was identified as the main contributor to T2D in Mexicans and accordingly, its frequency was higher as compared to other populations (f risk allele G: 34.4% in this study, 1.7% in Europeans, 0.3% in Africans and 10% in East Asians). Interestingly, we found that rs75493593 is among the main associated variants with TG in Mexicans, in line with previous functionally in vitro studies [10,29].

Likewise, for HDLc levels, the most associated variant was the Native American private rs9282541 within *ABCA1* gene (f risk allele T: 0.11% in this study, 0% in Europeans, 0% in Africans and 0% in East Asians). It was followed by *LIPC* gene, which has shown to be the top one gene explaining the variation of HDLc levels in European population [30]. Regarding TC and LDLc levels, we found that *SPTLC3* and *ANXA9* had an effect on both traits.

Gender differences in the prevalence of dyslipidemias have been documented [31]. Similarly, sexually dimorphic *loci* having larger effect in women than in men, or vice versa, have been reported [32,33]. Accordingly, our findings suggest that the impact of genetic variation on the risk of developing dyslipidemias may depend on gender, given that the whole analyzed *loci* explained a higher percentage of the variance of TG and HDLc levels in men. In turn, they explained a higher percentage of the variance of TC levels in women.

In summary, although Mexican population was mainly originated from the admixture of European and Native American ancestral populations, our results suggest that an important proportion of the population contribution to dyslipidemias and T2D in Mexico is not fully explained by European common genetic variation but instead, it could be attributed to Native American specific variants. The above was demonstrated by the influence of *ABCA1* rs9282541 variant on HDLc levels, as well as by the role of *SLC16A11* rs75493593 variant on TG levels and T2D risk.

Regarding the GO biological processes involved, we found three of them enriched in genes associated with lipid levels in Mexican population: (i) lipid metabolism and homeostasis, (ii) organic hydroxy compound transport and, (iii) plasma lipoprotein particle organization. Accordingly, the most enriched KEGG pathways were: cholesterol metabolism, PPAR signaling and fat digestion and absorption. It is of interest that variants within genes that have been proved to be clinically and pharmacologically relevant for Caucasians were not among the most associated with lipid levels in Mexican population, including *PCSK9* and *PPARA*.

A potential limitation of this study is that the expanded sample size accounts for 3.6% of the Mexican adult population in the year 2000. However, the analyzed sample included individuals across all country. Importantly, in the present study, we used plasma glucose levels for the diagnosis of T2D as no HbA1c determinations were available. Although it is known that HbA1c levels are influenced by genetic variants, only a subset of them impacts blood glucose control, while the remaining are associated with the function, structure, and lifespan of the red blood itself. Such last group of variants might artificially lower or elevate HbA1c levels independently from glucose concentration and has been reported in high diabetes-risk minority groups, like Mexican [34,35].

An additional limitation of the present study resides in the number of genes and the examined genetic variants, as they were selected from previous published reports. We observed major differences in allelic frequencies for previously known variants. For instance, the risk allele frequencies of *LDLR* variant rs2228671 range from 1% to 10% in other populations, while in Mexicans it has a very low frequency (0.2%). Also, due to the rapid progress in this area, it is likely that we have missed the inclusion of the most recently identified genetic variants related to lipid variation.

Given our large Native American inheritance, it is likely that Mestizo populations, including Mexican Mestizos, will largely benefit from other genotyping strategies such as exome and whole genome sequencing. These strategies are expected to result in the identification of private Mexican genetic variation. Moreover, we are certain that our findings will promote the prioritization of candidate genes with the strongest effect on lipid traits, which may in turn, lead to the identification of additional therapeutic targets for the treatment of dyslipidemias in Mexico. As most GWAS originally focus on populations of European ancestry, it is likely that Native American-derived populations such as Mexicans carry additional new variants with potential larger effect sizes. Therefore, the study of admixed populations provides an opportunity to identify new population-specific variation that may greatly impact lipid levels.

## 5. Conclusions

In conclusion, our results support the idea that in Mexico, the known genetic risk variation for the development of several dyslipidemias and T2D, explain a low proportion of the total risk variance when analyzing a population-based nationwide sample. *GCKR*, *AKR1C4*, *HMGCR*, *SLC16A11*, *APOC3*, *APOA5/BUD13*, *ABCA1*, *LIPC*, *LPL* and *APOA1*, *LCAT*, *MLXIPL*, *RBM6*, *SPTLC3*, *ANXA9*, *LDLR*, *FADS1-2-3*. *NCAN* and *APOB* were among the most associated genes determining lipid levels in Mexican population. Remarkably, the analyzed *SLC16A11* and *ABCA1* variants, derived from Native American ancestry, showed the highest contribution to T2D and lipid levels in our population.

Even though the Mexican population mainly originated from the admixture of European and Native American ancestral populations, our results show that a score obtained from European-derived genetic variants do not completely capture the underlying genetic component for T2D and dyslipidemias in Mexican-mestizos.

Thus, our results emphasize the need to identify additional genetic risk variants, which are likely to be private for Mexican and other Native American-derived population. Such variants could potentially have higher effect sizes and its corresponding genes and pathways may represent new potential therapeutic targets.

## Figures and Tables

**Table 1:** Sample description.

Variable	Raw	Expansion
N	1627	1,920,663
Age (years)	38.21 ± 14.84	35.21 ± 13.97
Sex (% females)	32.49	50.0
Diabetic status (%)	13	8.0
Systolic blood pressure (mmHg)	120.81 ± 15.71	120.8 ± 14.99
Diastolic blood pressure (mmHg)	79.51 ± 11.4	79.10 ± 11.36
BMI (kg/m^2^)	27.18 ± 5.38	26.76 ± 5.20
Waist circumference (cm)	93.26 ± 15.7	92.16 ± 15.85
Glucose (mg/dL)	74 [65–86]	74 [65–85]
Creatinine (mg/dL)	0.9 [0.8–1]	0.9 [0.8–1.1]
Total cholesterol (mg/dL)	190 [164–223]	183 [159–219]
Triglycerides (mg/dL)	146 [98–212]	131 [87–192]
HDL cholesterol (mg/dL)	43 [37–52]	41 [34–48]
LDL cholesterol (mg/dL)	114 [94–139]	111 [94–136]
Native American ancestry (%)	61.9 [49.1–77.3]	62.9 [50.4–76.8]

Mean ± sd, percentage or median [25–75th percentiles] calculated using both raw and expanded data. BMI: body mass index.

**Table 2:** Sample description stratified by diabetes and dyslipidemia status.

Variable	Euglycemia	Diabetes	Normolipidemia	Dyslipidemia
N	1,095,837	95,177	643,776	1,271,329
Age (years)	34.17 ± 13.45	50.30 ± 14.85 **^a^**	31.48 ± 13.06	37.06 ± 14.01 **^b^**
Sex (% females)	48.3	68.5 **^a^**	59.85	45.03 **^b^**
Diabetic status (%)	0	100	6.0	8.9
Dyslipidemia status (%)	70.6	78.7	0	100
Systolic blood pressure (mmHg)	120.9 ± 14.99	129.4 ± 17.67 **^a^**	116.22 ± 12.25	123.14 ± 15.73 **^b^**
Diastolic blood pressure (mmHg)	79.10 ± 14.96	81.87 ± 11.97	76.17 ± 9.32	80.62 ± 12 **^b^**
BMI (kg/m^2^)	26.79 ± 5.36	29.43 ± 4.98 **^a^**	25.26 ± 4.99	27.53 ± 5.1 **^b^**
Waist circumference (cm)	92.75 ± 16.33	101.19 ± 14 **^a^**	87.08 ± 14.50	94.71 ± 15.9 **^b^**
Glucose (mg/dL)	75 [66–85]	190 [136–255] **^a^**	72 [63–82]	75 [67–87] **^b^**
Creatinine (mg/dL)	0.9 [0.8–1.1]	0.9 [0.8–1.0]	0.9 [0.8–1.0]	0.95 [0.8–1.1] **^b^**
Total cholesterol (mg/dL)	187 [161–222]	200 [176–242.3] **^a^**	170 [154.6–182]	207 [166–232.1] **^b^**
Triglycerides (mg/dL)	144 [97–207]	188.2 [139.1–243.5] **^a^**	94 [72–116.9]	175 [125–238] **^b^**
HDL cholesterol (mg/dL)	41 [34–48]	40 [33–45]	46 [40–53]	40 [32–48] **^b^**
LDL cholesterol (mg/dL)	112.3 [96–140]	124.5 [107–152.7]	102 [89–114]	127 [101–150] **^b^**
Native American ancestry (%)	58.2 [47–72.8]	64.8 [53.9–80.4] **^a^**	64.5 [53.2–81.4]	61.5 [49.5–76]

Mean ± sd, percentage or median [25–75th percentiles] calculated using both raw and expanded data. Expanded data is only displayed. Diabetes is defined as American Diabetes Association criteria. Dyslipidemia is defined as TC levels ≥200 mg/dL or TG levels ≥160 mg/dL or HDLc levels <35 mg/dL. ^a^ *p* value < 0.05 from a survey *t* test or a survey rank sum comparison between normoglycemic versus diabetic subjects. ^b^ *p* value < 0.05 from a survey *t* test or a survey rank sum comparison between normolipidemic versus dyslipidemic subjects. BMI: body mass index.

## Supplementary Materials

The following are available online at https://www.mdpi.com/2073-4425/11/1/114/s1, Figure S1: Estimation of proportions of the variance explained by genetic and environmental factors, Figure S2: Variance explained by previously known genes associated to dyslipidemias according to gender, Figure S3: Functional role of the genes analyzed in this study, Table S1: List of SNPs genotyped in this study, Table S2: Association analyses results for lipid traits, Table S3: Association analyses results for T2D risk.
