# Supplementary material for: Contribution of Known Genetic Risk Variants to Dyslipidemias and Type 2 Diabetes in Mexico: A Population-Based Nationwide Study
Source: Genes (Basel). 2020 Jan 20;11(1):114. doi: 10.3390/genes11010114 (PMC7016795; doi:10.3390/genes11010114)
Supplement: Supplementary file 1 [file genes-11-00114-s001.pdf]

Supplementary Materials

Model 1: trait ~rsPS

Model 2: trait ~rsPS + sex + age + BMI + ancestry (+ diabetic status)

Total genetic variance = r^2 model 1

Environmental variance = r^2 (model 2 – model 1)

Figure S1. Genetic and environmental variances estimation. rsPS: restricted-to-significant polygenic score.

Table S1. List of SNPs genotyped in this study.

	Trait	SNP	Minor allele	Nearest gene	Minor allele frequency				
					Mexico	AFR	EAS	EUR	SAS
1	Lipids	rs10102164	A	*SOX17*	0.123	0.173	0.207	0.173	0.234
2	Lipids	rs10401969	C	*CILP2*	0.042	0.181	0.107	0.071	0.117
3	Lipids	rs10490626	A	*INSIG2*	0.028	0.001	0	0.080	0.044
4	Lipids	rs1077835	T	*LIPC*	0.337	0.427	0.581	0.786	0.686
5	Lipids	rs10889337	A	*DOCK7*	0.445	0.580	0.232	0.312	0.479
6	Lipids	rs1121980	T	*FTO*	0.234	0.467	0.211	0.443	0.387
7	Lipids	rs12255372	T	*TCF7L2*	0.114	0.302	0.010	0.292	0.221
8	Lipids	rs1260326	T	*GCKR*	0.310	0.094	0.481	0.411	0.200
9	Lipids	rs12678919	G	*LPL*	0.034	0.120	0.120	0.130	0.080
10	Lipids	rs12748152	T	*NR0B2*	0.018	0.008	0.029	0.073	0.048
11	Lipids	rs12916	C	*HMGCR*	0.372	0.235	0.511	0.413	0.558
12	Lipids	rs1349411	C	*RP11-185H22.1*	0.296	0.540	0.550	0.230	NA
13	Lipids	rs138326449	A	*APOC3*	0	0	0.001	0.003	0
14	Lipids	rs1424032	G	*Intergenic*	0.259	0.064	0.205	0.202	0.116
15	Lipids	rs1532624	T	*CETP*	0.372	0.104	0.291	0.426	0.480
16	Lipids	rs16942887	A	*LCAT*	0.170	0.247	0.026	0.134	0.171
17	Lipids	rs174546	C	*FADS1-2-3*	0.269	0.978	0.434	0.653	0.863
18	Lipids	rs17695224	A	*FPR3*	0.340	0.139	0.205	0.254	0.227
19	Lipids	rs1800961	T	*HNF4A*	0.038	0.001	0.016	0.037	0.029
20	Lipids	rs1832007	G	*AKR1C4*	0.222	0.025	0.111	0.120	0.109
21	Lipids	rs2000999	A	*HPR*	0.171	0.055	0.310	0.192	0.433

22	Lipids	rs2013208	C	*RBM6*	0.226	0.402	0.144	0.506	0.211
23	Lipids	rs2030746	T	*LOC84931*	0.370	0.534	0.496	0.401	0.374
24	Lipids	rs2036402	G	*HAVCR1*	0.450	0.041	0.123	0.291	0.204
25	Lipids	rs2038037	A	*MRPL42P2*	0.228	0.085	0.072	0.560	0.369
26	Lipids	rs2070665	T	*APOA1*	0.209	0.120	0.352	0.106	0.338
27	Lipids	rs2131925	G	*ANGPTL3*	0.448	0.692	0.232	0.305	0.469
28	Lipids	rs2228603	T	*NCAN*	0.017	0.006	0.056	0.068	0.074
29	Lipids	rs2228671	T	*LDLR*	0.002	0.023	0.013	0.101	0.074
30	Lipids	rs2278426	A	*DOCK6*	0.300	0.202	0.232	0.043	0.147
31	Lipids	rs2286276	A	*MLXIPL*	0.115	0.339	0.093	0.255	0.156
32	Lipids	rs2287623	A	*ABCB11*	0.375	0.554	0.740	0.604	0.603
33	Lipids	rs2479409	A	*PCSK9*	0.268	0.748	0.331	0.680	0.721
34	Lipids	rs267738	G	*ANXA9*	0.070	0.006	0.026	0.191	0.050
35	Lipids	rs2710642	G	*EHBP1*	0.337	0.089	0.270	0.375	0.263
36	Lipids	rs2954029	T	*TRIB1*	0.342	0.291	0.552	0.448	0.351
37	Lipids	rs314253	T	*DLG4*	0.450	0.615	0.548	0.666	0.769
38	Lipids	rs364585	A	*SPTLC3*	0.357	0.130	0.434	0.363	0.309
39	Lipids	rs3780181	G	*VLDLR*	0.104	0.246	0.113	0.053	0.071
40	Lipids	rs4142995	T	*SNX13*	0.461	0.798	0.496	0.398	0.463
41	Lipids	rs4149310	A	*ABCA1*	0.395	0.244	0.281	0.842	0.803
42	Lipids	rs4253772	T	*PPARA*	0.049	0.008	0.001	0.119	0.037
43	Lipids	rs4299376	G	*ABCG5/ABCG8*	0.106	0.140	0.005	0.307	0.219
44	Lipids	rs4420638	G	*APOE*	0.087	0.220	0.099	0.198	0.102
45	Lipids	rs4520	C	*APOC3*	0.480	0.720	0.396	0.705	0.546
46	Lipids	rs4530754	A	*CSNK1G3*	0.393	0.819	0.343	0.584	0.420
47	Lipids	rs4650994	A	*ANGPTL1*	0.380	0.123	0.536	0.463	0.515

48	Lipids	rs4722551	C	*MIR148A*	0.175	0.023	0.019	0.172	0.099
49	Lipids	rs4846914	G	*GALNT2*	0.421	0.943	0.771	0.399	0.605
50	Lipids	rs4942486	T	*BRCA2*	0.489	0.489	0.467	0.484	0.532
51	Lipids	rs5128	C	*APOC3*	0.220	0.835	0.663	0.876	0.662
52	Lipids	rs5167	G	*APOC2*	0.503	0.457	0.572	0.367	0.342
53	Lipids	rs540796	A	*PCSK9*	0.079	0.225	0.012	0.179	0.094
54	Lipids	rs6065906	C	*PLTP*	0.069	0.163	0.026	0.204	0.250
55	Lipids	rs629301	C	*SORT1*	0.209	0.399	0.047	0.214	0.260
56	Lipids	rs635634	A	*ABO*	0.104	0.086	0.187	0.185	0.132
57	Lipids	rs6511720	T	*LDLR*	0.051	0.147	0.012	0.110	0.079
58	Lipids	rs6831256	A	*LRPAP1*	0.432	0.332	0.619	0.602	0.513
59	Lipids	rs6882076	T	*TIMD4*	0.156	0.685	0.273	0.353	0.305
60	Lipids	rs702485	A	*DAGLB*	0.237	0.186	0.106	0.565	0.342
61	Lipids	rs7241918	G	*LIPG*	0.042	0.024	0.115	0.163	0.137
62	Lipids	rs7248104	A	*INSR*	0.335	0.269	0.300	0.422	0.410
63	Lipids	rs731839	G	*PEPD*	0.467	0.371	0.571	0.333	0.626
64	Lipids	rs75493593 *	T	*SLC16A11*	0.344	0.003	0.100	0.017	0.004
65	Lipids	rs75557067	A	*TMEM161*	0.002	0.030	0	0	0
66	Lipids	rs7575840	T	*APOB*	0.307	0.116	0.117	0.322	0.126
67	Lipids	rs7903146 Ψ	T	*TCF7L2*	0.168	0.260	0.023	0.317	0.299
68	Lipids	rs8077889	C	*MPP3*	0.069	0.171	0.001	0.253	0.162
69	Lipids	rs838880	C	*SCARB1*	0.409	0.751	0.558	0.313	0.450
70	Lipids	rs9282541*	T	*ABCA1*	0.114	0.001	0	0	0
71	Lipids	rs9305020	C	*LDLR*	0.216	0.728	0.041	0.175	0.140
72	Lipids	rs964184	G	*APOA5/BUD13*	0.357	0.221	0.240	0.162	0.229
73	Lipids	rs9949617	T	*TMEM241*	0.411	0.345	0.159	0.168	0.172

74	Lipids	rs998584	C	*VEGFA*	0.396	0.855	0.432	0.496	0.313
75	Lipids	rs9987289	A	*PPP1R3B*	0.279	0.189	0.012	0.075	0.092
76	T2D	rs10811661	C	*CDKN2A/2B*	0.101	0.045	0.436	0.168	0.132
77	T2D	rs1111875	T	*HHEX*	0.381	0.184	0.716	0.425	0.644
78	T2D	rs11717195	C	*ADCY5*	0.360	0.088	0.004	0.174	0.189
79	T2D	rs1359790	T	*SPRY2*	0.431	0.080	0.278	0.267	0.158
80	T2D	rs149483638 *	T	*IGF2*	0.224	0	0.010	0.001	0
81	T2D	rs1552224	G	*CENTD2*	0.057	0.008	0.081	0.150	0.200
82	T2D	rs1801282	G	*PPARG*	0.119	0.005	0.026	0.120	0.120
83	T2D	rs2237897	T	*KCNQ1*	0.309	0.081	0.353	0.047	0.013
84	T2D	rs243021	C	*BCL11A*	0.391	0.607	0.348	0.553	0.508
85	T2D	rs340874	G	*PROX1*	0.357	0.092	0.420	0.531	0.531
86	T2D	rs3802177	T	*SLC30A8*	0.261	0.076	0.461	0.283	0.254
87	T2D	rs4402960	T	*IGF2BP2*	0.204	0.567	0.249	0.304	0.455
88	T2D	rs4458523	T	*WFS1*	0.257	0.420	0.096	0.368	0.334
89	T2D	rs483353044 *		*HNF1A*	0	0	0	0	0
90	T2D	rs459193	T	*ANKRD55*	0.230	0.474	0.512	0.291	0.357
91	T2D	rs516946	A	*ANK1*	0.168	0.207	0.145	0.231	0.190
92	T2D	rs5219	T	*KCNJ11*	0.399	0.023	0.338	0.353	0.396
93	T2D	rs6815464	G	*MAEA*	0.409	0.123	0.425	0.026	0.167
94	T2D	rs7041847	G	*GLIS3*	0.374	0.046	0.499	0.467	0.397
95	T2D	rs7178572	A	*HMG20A*	0.380	0.506	0.629	0.307	0.516
96	T2D	rs75493593 *	T	*SLC16A11*	0.345	0.003	0.100	0.017	0.004
97	T2D	rs7578597	C	*THADA*	0.044	0.309	0.006	0.091	0.132
98	T2D	rs7756992	G	*CDKAL1*	0.349	0.633	0.472	0.281	0.269
99	T2D	rs7903146	T	*TCF7L2*	0.169	0.260	0.023	0.317	0.299

100	T2D	rs7961581	C	*TSPAN8/LGR5*	0.122	0.176	0.217	0.298	0.312
101	T2D	rs824248	T	*LINGO2*	0.334	0.202	0.415	0.237	0.259
102	T2D	rs849135	A	*JAZF1*	0.331	0.211	0.013	0.503	0.213
103	T2D	rs9936385	C	*FTO*	0.180	0.494	0.169	0.414	0.289

[a] Single Nucleotide Polymorphisms (SNPs) are ordered by rs identifier.
[b] * means Native American-private risk genetic variants.
[c] means SNPs excluded from analyses given their low variation in Mexican people.
[d] Ψ means SNP excluded from analyses given their LD with another SNP
[e] Weighted allele frequencies for Mexico were computed using contingency tables for survey data

Table S2. Association analyses results for lipid traits.

	Gene	Tag SNP	TG		TC		HDLc		LDLc	
			B	*p* value	B	*p* value	B	*p* value	B	*p* value
1	*ABCA1*	rs9282541 *	-1095.4	0.196	95.9	0.402	42.3	**2.6E-04**	37.2	0.737
2	*ABCA1*	rs4149310	-225.3	0.443	-22.3	0.699	-19.5	0.275	-69.6	0.644
3	*ABCB11*	rs2287623	-217.9	0.547	-83.0	0.782	-18.2	0.878	-96.6	0.567
4	*ABCG5/ABCG8*	rs4299376	15587.4	0.738	211.4	0.356	-52.6	0.191	54.2	0.729
5	*ABO*	rs635634	-151.6	0.665	101.9	0.126	-102.8	0.287	85.1	0.172
6	*AKR1C4*	rs1832007	5539.4	**0.005**	-306.4	0.384	-4.7	0.884	-190.7	0.221
7	*ANGPTL1*	rs4650994	899.0	0.069	-44.3	0.894	28.1	0.548	-51.0	0.853
8	*ANGPTL3*	rs2131925	49.4	0.720	-65.2	0.682	29.1	0.638	-418.3	0.219
9	*ANXA9*	rs267738	-3070.4	0.101	-213.2	**0.025**	92.9	0.248	-267.3	**0.007**
10	*APOA1*	rs2070665	160.5	0.067	-152.4	0.244	42.8	**0.019**	178.4	0.486
11	*APOA5/BUD13*	rs964184	126.2	**0.043**	-23.6	0.604	33.2	0.076	-34.2	0.592
12	*APOB*	rs7575840	-1367.2	0.240	135.2	0.190	-390.7	0.156	93.9	**0.041**
13	*APOC2*	rs5167	353.9	0.883	149.2	0.242	-107.5	0.055	-9.9	0.822

15	*APOC3*	rs5128	-156.4	0.067	173.1	0.074	-45.4	**0.011**	-135.9	0.575
14	*APOC3*	rs4520	201.0	**0.034**	-93.8	0.597	59.8	0.054	-16.3	0.914
16	*APOE*	rs4420638	317.6	0.259	132.8	0.217	106.8	0.280	75.6	0.617
17	*BRCA2*	rs4942486	-16.2	0.960	447.4	0.174	65.3	0.057	146.2	0.502
18	*CETP*	rs1532624	98.7	0.682	85.9	0.174	13.8	0.109	-41.5	0.869
19	*CILP2*	rs10401969	-159.4	0.383	-90.9	0.227	226.9	0.181	7.2	0.918
20	*CSNK1G3*	rs4530754	-541.8	0.423	302.1	0.898	-20.6	0.772	-1320.4	0.364
21	*DAGLB*	rs702485	1653.0	0.266	-395.5	0.315	-7.0	0.989	-307.6	0.434
22	*DLG4*	rs314253	43.7	0.982	126.7	0.146	-30.4	0.460	568.8	0.192
23	*DOCK6*	rs2278426	512.4	0.334	5.7	0.911	7.2	0.608	32.8	0.595
24	*DOCK7*	rs10889337	63.2	0.650	-81.2	0.653	26.8	0.644	-654.0	0.162
25	*EHBP1*	rs2710642	38.3	0.978	60.5	0.564	15.1	0.701	20.1	0.702
26	*FADS1-2-3*	rs174546	66.3	0.845	-596.4	0.242	519.5	0.176	-498.5	0.018
27	*FPR3*	rs17695224	-2467.4	0.186	7.9	0.990	-174.1	0.429	-7.4	0.938
28	*FTO*	rs1121980	-1438.0	0.052	-828.2	0.346	38.0	0.884	-119.1	0.136
29	*GALNT2*	rs4846914	232.4	0.383	-56.8	0.593	14.9	0.548	-77.1	0.523
30	*GCKR*	rs1260326	441.3	**2.6E-05**	-19.9	0.873	293.4	0.056	97.0	0.643
31	*HAVCR1*	rs2036402	990.9	0.982	-65.8	0.863	-114.9	0.873	-190.2	0.060
32	*HMGCR*	rs12916	-412.8	**0.019**	-111.3	0.536	323.0	0.668	-1.7	0.995
33	*HNF4A*	rs1800961	-966.9	0.765	36.5	0.593	19.6	0.497	43.5	0.382
34	*HPR*	rs2000999	3332.9	0.321	435.1	0.238	-44.9	0.688	-135.6	0.213
35	*INSIG2*	rs10490626	1231.1	0.248	-85.6	0.540	-61.2	0.697	-84.7	0.340
36	*INSR*	rs7248104	-178.4	0.397	-137.4	0.214	292.8	0.146	-6.5	0.958
37	*Intergenic*	rs1424032	2484.0	0.181	-58.6	0.918	-4796.3	**0.008**	-46.6	0.954
38	*LCAT*	rs16942887	-233.0	0.798	189.5	0.670	-50.8	**0.022**	-60.7	0.722
40	*LDLR*	rs9305020	-230.4	0.642	268.1	0.105	-27.0	0.460	196.1	**0.015**

39	*LDLR*	rs6511720	-4419.2	0.540	-64.6	0.383	83.1	0.395	-4.6	0.914
41	*LIPC*	rs1077835	-412.9	0.698	325.6	0.505	45.7	**0.001**	-20.1	0.954
42	*LIPG*	rs7241918	-1871.8	0.207	39.7	0.844	30.9	0.569	55.6	0.556
43	*LOC84931*	rs2030746	-19.5	0.993	283.7	0.190	170.9	0.257	622.5	**0.048**
44	*LPL*	rs12678919	-104.7	0.442	-3829.7	0.459	36.6	**0.017**	-733.1	0.116
45	*LRPAP1*	rs6831256	-2135.2	0.134	89.7	0.565	171.3	0.573	49.1	0.528
46	*MIR148A*	rs4722551	-288.1	0.372	-288.7	0.709	-53.1	0.630	-96.6	0.851
47	*MLXIPL*	rs2286276	59.4	0.706	-367.0	0.152	-284.7	**0.033**	100.0	0.533
48	*MPP3*	rs8077889	151.8	0.487	-253.8	0.298	77.7	0.064	-181.3	0.258
49	*MRPL42P2*	rs2038037	523.6	0.390	348.7	0.096	7.8	0.883	-1609.3	0.054
50	*NCAN*	rs2228603	-153.0	0.857	340.7	0.555	6.9	0.924	1023.9	**0.021**
51	*NR0B2*	rs12748152	65.7	0.842	-219.8	0.483	-47.5	0.711	-244.3	0.227
52	*PCSK9*	rs2479409	-678.7	0.799	-238.4	0.161	-114.2	0.453	-196.3	0.104
53	*PCSK9*	rs540796	-452.3	0.785	644.2	0.872	124.8	0.225	144.6	0.729
54	*PEPD*	rs731839	243.6	0.731	1457283.2	0.242	-1.2	0.985	53.3	0.848
55	*PLTP*	rs6065906	-238.7	0.497	-144.2	0.412	1406.8	0.214	444.0	0.648
56	*PPARA*	rs4253772	-1.2	0.998	-666.3	0.708	19.6	0.901	132.1	0.955
57	*PPP1R3B*	rs9987289	348.1	0.738	-20.6	0.716	-1.2	0.945	62.4	0.381
58	*RBM6*	rs2013208	-152.7	0.670	-196.6	0.276	-230.9	**0.037**	-245.5	0.200
59	*RP11-185H22.1*	rs1349411	-579.1	0.859	-471.1	0.532	37.9	0.664	110.9	0.385
60	*SCARB1*	rs838880	53.3	0.827	104.6	0.443	19.8	0.887	209.7	0.270
61	*SLC16A11*	rs75493593 *	2928.0	**0.034**	140.1	0.443	54.8	0.703	-33.1	0.908
62	*SNX13*	rs4142995	101.0	0.935	2239.5	0.118	-87.5	0.078	-514.1	0.129
63	*SORT1*	rs629301	323.5	0.517	57.6	0.365	31.3	0.728	23.7	0.551
64	*SOX17*	rs10102164	-3425.3	0.596	151.0	0.341	91.8	0.114	122.2	0.308
65	*SPTLC3*	rs364585	1591.1	0.326	87.2	**0.025**	-26.0	0.750	155.0	**0.024**

66	*TCF7L2*	rs12255372	-685.3	0.768	301.7	0.274	445.6	0.198	138.8	0.354
67	*TIMD4*	rs6882076	-30447.9	0.062	162.3	0.249	-1006.5	0.205	72.5	0.156
68	*TMEM241*	rs9949617	-562.9	0.249	-408.4	0.110	-1520.1	0.630	113.6	0.617
69	*TRIB1*	rs2954029	210.2	0.180	54.5	0.378	25.7	0.522	104.1	0.699
70	*VEGFA*	rs998584	479.4	0.258	145.7	0.755	37.2	0.248	56.6	0.529
71	*VLDLR*	rs3780181	-577.0	0.319	57.8	0.968	-525.5	0.086	6900.8	0.719

[a] Single Nucleotide Polymorphisms (SNPs) are ordered by gene name.

[b] * means Native American-private risk genetic variants.

Table S3. Association analyses results for type 2 diabetes risk.

	Gene	Tag SNP	OR	*p* value
1	*ADCY5*	rs11717195	0.978	0.921
2	*ANK1*	rs516946	1.272	0.270
3	*ANKRD55*	rs459193	1.058	0.874
4	*BCL11A*	rs243021	1.188	0.612
5	*CDKAL1*	rs7756992	1.747	0.189
6	*CDKN2A/2B*	rs10811661	0.451	0.210
7	*CENTD2*	rs1552224	0.613	0.370
8	*FTO*	rs9936385	1.316	0.413
9	*GLIS3*	rs7041847	0.966	0.924
10	*HHEX*	rs1111875	0.679	0.449
11	*HMG20A*	rs7178572	0.907	0.783
12	*IGF2*	rs149483638 *	1.216	0.092
13	*IGF2BP2*	rs4402960	1.014	0.952
14	*JAZF1*	rs849135	1.392	0.071
15	*KCNJ11*	rs5219	0.697	0.182
16	*KCNQ1*	rs2237897	1.010	0.933
17	*LINGO2*	rs824248	0.882	0.398
18	*MAEA*	rs6815464	0.910	0.649
19	*PPARG*	rs1801282	1.084	0.844
20	*PROX1*	rs340874	0.870	0.635
21	*SLC16A11*	rs75493593 *	1.340	**0.024**
22	*SLC30A8*	rs3802177	1.287	0.258
23	*SPRY2*	rs1359790	1.012	0.950
24	*TCF7L2*	rs7903146	1.126	0.616
25	*THADA*	rs7578597	1.000	1.000
26	*TSPAN8/LGR5*	rs7961581	0.908	0.638
27	*WFS1*	rs4458523	1.178	0.536

[a] Single Nucleotide Polymorphisms (SNPs) are ordered by gene name.

[b] * means Native American-private risk genetic variants.

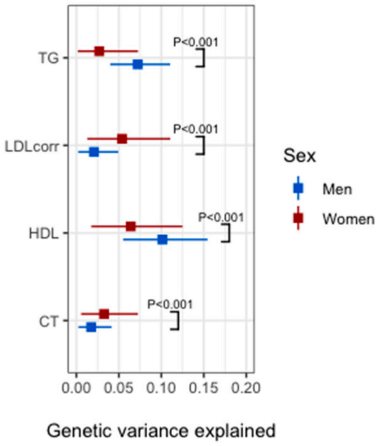

Trait	Sex	Rsq [95% CI]
TG	Men	0.072 [0.040-0.110]
TG	Women	0.027 [0.002-0.073]
LDLc	Men	0.021 [0.002-0.049]
LDLc	Women	0.054 [0.013-0.111]
HDLc	Men	0.101 [0.055-0.154]
HDLc	Women	0.064 [0.018-0.125]
TC	Men	0.017 [0.002-0.041]
TC	Women	0.033 [0.006-0.073]

Figure S2. Variance explained by previously known genes associated to dyslipidemias according to gender. It is shown the bootstrapped 95% confidence interval for r^2 based on 1,000 replications of regression models for complex surveys including either women or men only.

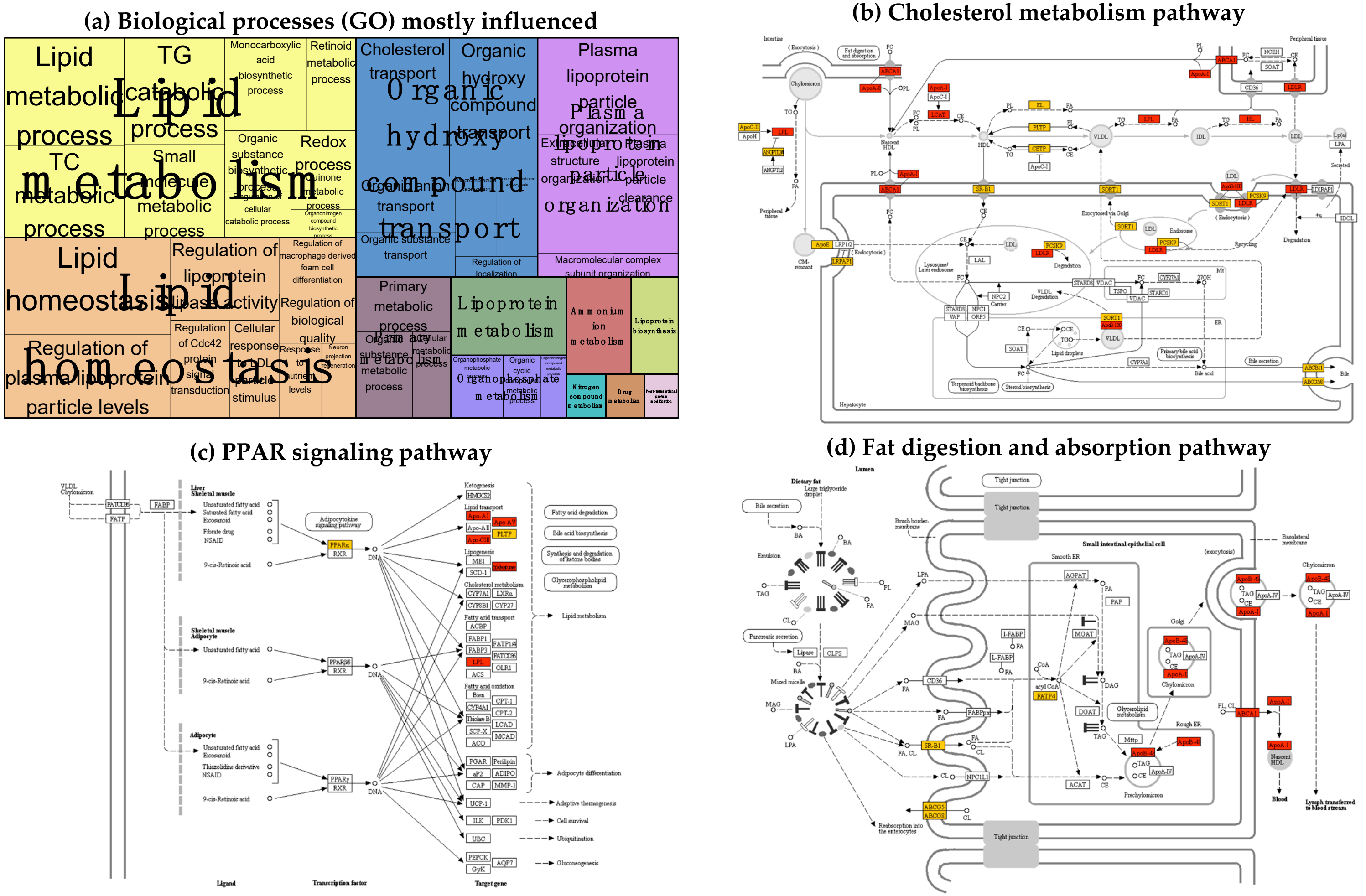

Figure S3. Functional role of the genes analyzed in this study. (**a**) Tree map of the biological processes mostly influenced the genes associated with lipid levels in Mexican population. (**b-d**) KEGG pathways enriched by the genes associated with lipid levels in Mexican population. Red squares highlight genes with an association *p* value < 0.05. Yellow squares highlight genes with an association *p* value ≥0.05. White squares include genes that were not analyzed in this study.
